# Clinicopathological significance of nuclear factor (erythroid-2)-related factor 2 (Nrf2) expression in gastric cancer

**DOI:** 10.1186/s12885-015-1008-4

**Published:** 2015-01-15

**Authors:** Yota Kawasaki, Sumiya Ishigami, Takaaki Arigami, Yoshikazu Uenosono, Shigehiro Yanagita, Yasuto Uchikado, Yoshiaki Kita, Yuka Nishizono, Hiroshi Okumura, Akihiro Nakajo, Yuko Kijima, Kosei Maemura, Shoji Natsugoe

**Affiliations:** Department of Digestive Surgery, Breast and Thyroid surgery Graduate School of Medical Sciences, Kagoshima University, Sakuragaoka 8-35-1, Kagoshima, 890-8520 Japan

**Keywords:** NF-E2-related factor 2, Gastric cancer, Antioxidants

## Abstract

**Background:**

The transcription factor nuclear factor (erythroid-2)–related factor 2 (Nrf2) was originally identified as a critical regulator of intracellular anti-oxidants and of phase II detoxification enzymes through its transcriptional up-regulation of many anti-oxidant response element (ARE)-containing genes. Nrf2 protects not only normal cells but also cancer cells from cellular stress, and enhances cancer cell survival. Some studies have shown that Nrf2 expression in cancer patients has clinical significance. However, there has been no comprehensive analysis of the nuclear expression level of Nrf2 in gastrointestinal cancer cells. In this study we aimed to immunohistochemically evaluate the expression of Nrf2, and to assess its clinical significance in gastric cancer.

**Methods:**

A total of 175 gastric cancer patients who received R0 gastrectomy with standard lymph node dissection were enrolled. We immunohistochemically evaluated Nrf2 expression in the paraffin-embedded surgically resected specimens of these 175 patients. Group differences were analyzed using the *χ*^2^ test, Fisher’s exact test, and the Mann–Whitney *U* test. Associations between Nrf2 expression and clinicopathological features, including clinical outcome, were assessed using univariate and multivariate analyses, and Kaplan-Meier curves with the log-rank test, respectively.

**Results:**

Nrf2 immunoreactivity was predominantly identified in the nucleus of gastric cancer cells. Nrf2 positivity was closely associated with tumor size, tumor depth, lymph node metastases, lymphovascular invasion, histology and stage (p < 0.05 for all). A log-rank test indicated that the overall survival of the Nrf2-positive group was significantly poorer than that of the Nrf2-negative group (p < 0.01). And, positive Nrf2 expression was significantly associated with resistance to 5FU-based adjuvant chemotherapy (p = 0.024).

**Conclusions:**

Nrf2 expression was positively associated with aggressive tumor behavior in gastric cancer. This result suggests that Nrf2 expression in gastric cancer is a potential indicator of worse prognosis.

## Background

Gastric cancer is the fourth-most common gastrointestinal cancer and the second-most common fatal gastrointestinal cancer in the world. East Asian countries including Japan are among the highest-risk areas for gastric cancer [1,2]. In Japan, the establishment of mass screening using photo fluoroscopy or gastrointestinal fibroscopy has led to gastric cancer being diagnosed at early stages. A favorable prognosis is expected for these gastric cancer patients following curative surgery or endoscopic treatment. However, some patients show an unfavorable course despite receiving curative resection. The postoperative outcome of gastric cancer strongly depends on TNM factors [3,4]. In addition to these critical clinicopathological factors, the prognosis of gastric carcinoma is influenced by several biological variables. In this context, it is necessary to find novel cancer-related factors that can be used as markers for the diagnosis and treatment of gastric cancer. It is generally accepted that oxidative stress (OS) is involved in the pathophysiology of degenerative diseases. Moreover, the generation and increase of OS and secondary DNA oxidative damage are also known to be related to the damage and malignant transformation of gastric mucosa [5]. Thus, OS has been considered to play important roles in the carcinogenesis of gastric cancer [5,6].

The transcription factor nuclear factor (erythroid-2)–related factor 2 (Nrf2), which is a basic redox-sensitive bZIP transcription factor, was originally identified as a critical regulator of intracellular antioxidants and of phase II detoxification enzymes through the transcriptional upregulation of many antioxidant response element (ARE) -containing genes [7-9] . Under basal conditions, Nrf2 is bound to the kelch-like ECH-associated protein 1 (Keap1), a cul3-based E3 ubiquitin ligase adapter that regulates Nrf2 ubiquitination and proteasome-dependent degradation [10].

Upon exposure of cells to oxidative stress or chemopreventive compounds, Nrf2 translocates to the nucleus, forms a heterodimer with its obligatory partner Maf, binds to the ARE sequences in DNA and activates the transcription of downstream genes such as antioxidants and phase II detoxification enzymes [8,11-13]. The important biological role of Nrf2 is underlined by findings demonstrating Nrf2-dependent protection against many human diseases or pathological states such as cancer, neurodegenerative diseases, cardiovascular disease, inflammation, pulmonary fibrosis, acute pulmonary injury and Nrf2 also slows the process of aging [14-18]. Thus Nrf2 has been considered as a “good” transcription factor that is essential for protection against oxidative stress [19]. However, emerging data has revealed the dark side of Nrf2 [20]. It has recently been shown that aberrant activation of the Nrf2 pathway occurs frequently in cancer cells and tumor tissues. Nrf2 protects not only normal cells from transforming into cancer cells, but also protects cancer cells from cellular stress, enhances cancer cell survival and is associated with cancer growth and progression [18]. Since these revelations, there has been no comprehensive analysis of the expression level of Nrf2 in the nucleus of gastric cancer cells, and there have been no studies aimed at clarifying the relationship between the expression level of Nrf2 in the nucleus of gastric cancer cells and clinical outcome. In the current study, we carried out the first examination of the expression level of Nrf2 in the nucleus of gastric cancer cells and further discuss the clinical significance of Nrf2 expression with respect to anti- oxidative stress.

## Methods

### Detection of Nrf2 expression in gastric cancer cell lines by Western blot analysis

The gastric carcinoma cell lines MKN74, MNK45, KATO-III and NUGC4 were purchased from the Japanese Physical and Chemical Institute, Tokyo, Japan. The cells were incubated in RPMI 1640 with 10% fetal bovine serum (FBS), 100 units/ml penicillin, and 100 μg/ml streptomycin at 37°C in a cell incubator. All cells were harvested by centrifugation, rinsed with phosphate buffered saline (PBS) and total protein was extracted in lysis buffer. Nuclear extracts and cytoplasmic fractions for Western blotting of Nrf2 were prepared using a Nuclear/Cytosol Fractionation Kit (K266-25, BioVison, California,USA). Western blot analysis of Nrf2 expression in gastric cancer cell lines was performed as follows. Denatured protein extracted from the nucleus or cytoplasm was separated on an SDS-polyacrylamide gel and transferred to Hybond membrane, which was then blocked overnight in 5% skim milk in Tris-buffered saline (TBS). The membrane was then incubated with mouse monoclonal antibody against Nrf2 (sc-365949, Santa Cruz Biotechnology, Inc., Santa Cruz, CA, USA) at 1:500 dilution for overnight. The membrane was rinsed with TBS-Tween 20 (TBST) and incubated with anti-mouse IgG conjugated to horseradish peroxidase (HAF007, RD Syestems, Minneapolis, USA) for 15 minutes. Immunoreactive bands were visualized on X-ray film (Fuji, Japan) using ECL-Plus detection reagents (RPN2132, GE Healthcare, Buckinghamshire, England). Subsequently, to confirm nuclear and cytoplasmic fractions, the membrane was washed with WB Stripping Solution (Nakalai Tesque, Tokyo, Japan) for 15 minutes and re-probed for nuclear and cytoplasmic markers using the anti-Lamin B1 antibody (ab16048, Abcam, Cambridge, USA, 1:1000) and the anti-α-Tubulin antibody (CP06, Calbiochem, MA, USA, 1:1000) respectively.

### Patients and specimens

This study included 175 patients with gastric adenocarcinoma that had invaded into submucosal layer or deeper. All patients underwent curative gastrectomy with lymph node dissection at Kagoshima University Hospital from January 1995 to December 2004. Among 175 patients, 57, 14, 99, and 5 patients received distal, proximal, total and partial gastrectomy, respectively. The male to female ratio was 116 to 59, and the average age was 66 years, ranging from 31 to 84 years. The number of patients with stage I, II and III gastric cancers was 47, 33 and 95, respectively. Histopathologically, 71 cases were differentiated (papillary, well-differentiated and moderately differentiated tubular adenocarcinoma) and 104 were undifferentiated (poorly differentiated adenocarcinoma, mucinous adenocarcinoma and signet-ring cell carcinoma) carcinomas according to the 7^th^ edition of tumor node metastasis classification. None of these patients received chemotherapy before surgery. Written informed consent was obtained from the patients and the study was approved by the Ethical Committee of Kagoshima University Hospital (registration number 35–24). This investigation conformed to the principles outlined in the Declaration of Helsinki. Clinicopathological findings are described according to the 7^th^ edition of tumor node metastasis classification.

### Immunohistochemical analysis of Nrf2 in gastric cancer and its evaluation

Tumor samples were fixed with 10% formaldehyde in PBS, embedded in paraffin, sectioned into 4-μm thick sections and mounted on glass slides for immunohistochemical analysis. Sections were deparaffinised in xylene and dehydrated in a series of graded ethanol. The endogenous peroxidase activity of the specimens was blocked by immersing the slides in a 3% hydrogen peroxidase–methanol solution for 10 min at room temperature. After washing three times with PBS (5 min per wash), the sections were treated with 1% bovine serum albumin for 30 min at room temperature to block nonspecific reactions. For staining with anti-Nrf2 antibodies, sections were pretreated with citrate buffer for 10 min at 121°C in a microwave oven to retrieve antigenicity. The sections were washed three times with PBS for 5 min each time, and were then blocked by treatment with PBS containing 3% skim milk for 10 min at room temperature. The blocked sections were incubated with the diluted primary anti-Nrf2 antibody (sc-365949, Santa Cruz Biotechnology, Inc., 1:200,) in PBS at 4°C overnight, followed by staining with a streptavidin–biotin–peroxidase kit (Nichirei, Tokyo, Japan). The sections were washed three times in PBS for 5 min per wash, and the immune complex was visualized by incubating the sections with diaminobenzidine tetra hydrochloride. The sections were rinsed briefly in water, counterstained with hematoxylin, and mounted. Noncancerous placental tissue was used as the positive control for Nrf2. Nrf2 expression was determined by counting the number of cancer cells in which the nucleus was stained with the anti-Nrf2 antibody. All immunostained slides were evaluated by two independent observers (YK and SI), who were unaware of the clinical data and disease outcome. To evaluate Nrf2 expression, 10 representative fields within the tumor were selected, and expression in a total of 1000 cancer cells (100 cells per field) was evaluated using high power (×400) microscopy. In the case of coexistence of differentiated and undifferentiated area in same section, we evaluated the expression level of Nrf2 in the undifferentiated area, even though the area of undifferentiated is smaller than differentiated area. The average labeling index of Nrf2 was assessed according to the proportion of positive cells in each field. Expression of Nrf2 was assessed based on both the proportion of positive cells and the intensity of staining. The intensity of Nrf2 staining was quantified using a three-value intensity score (0, 1+, or 2+). The extent of reactivity was evaluated according to Solis et al. [21] and was expressed as a percentage (0-100%). Immunohistochemical scores, which ranged from 0 to 200, were calculated by multiplying the values of the intensity and the extent of reactivity. These scores were used to determine Nrf2 expression levels. Since the median score of Nrf2 expression in gastric cancer was 110 in the present study, a cut-off value of 100 was decided on.

### Association between the expression level of Nrf2 in gastric cancer and chemoresistance

To evaluate whether the expression level of Nrf2 in gastric cancer affects chemoresistance, we compared the expression level of Nrf2 in gastric cancer patients who received 5-Fluorouracil (5FU) based adjuvant chemotherapy. We defined “5FU resistance” as gastric cancer that recurred after adjuvant chemotherapy. As a standard protocol, we administer 5FU-based adjuvant chemotherapy to patients with stage II or III cancer. We excluded patients who were not able to continue adjuvant chemotherapy due to side effects. As a result, 72 patients were enrolled in this analysis.

### Statistical analysis

Statistical analysis of group differences was performed using the *χ*^2^ test, Fisher’s exact test and the Mann–Whitney *U* test. The Kaplan-Meier method was used for survival analysis, and differences in survival were estimated by using the log-rank test. Prognostic factors were examined by univariate and multivariate analyses (Cox proportional hazards regression model). A *p* value < 0.05 was considered to indicate statistical significance. All statistical analyses were performed using the StatFlex version 6.0 for Windows software (StatFlex version 6.0; Artec Inc., Osaka, Japan).

## Results

### Nrf2 expression in gastric carcinoma cell lines

**Figure 1:**
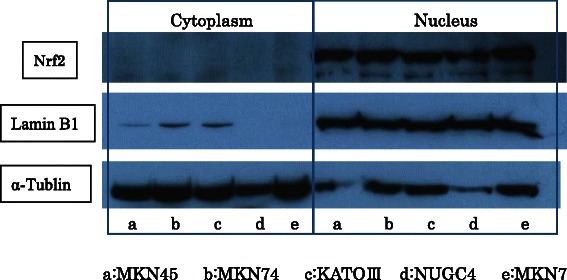
**Nuclear factor (erythroid-2)–related factor 2(Nrf2) expression in gastric carcinoma cell lines.** Nrf2 protein expression in nuclear and cytoplasmic extracts of the gastric cell lines MKN74, MNK45, KATO-III, and NUGC4 was assayed by Western blotting. The blot was re-probed with anti-Lamin B1 and anti-α-Tubulin antibodies that were used as positive controls for the nucleus and cytoplasm, respectively. Nuclear and cytoplasmic fractions were clearly separated. The Nrf2 protein was identified mainly in the nucleus of all gastric cancer cell lines. Little Nrf2 was detected in the cytoplasm.

The expression of Nrf2 in nuclear and cytoplasmic fractions of gastric carcinoma cell lines was analyzed by Western blotting. Nrf2 was expressed mainly in the nucleus of all of the gastric cancer cell lines and little expression was observed in the cytoplasm (Figure 1).

### Nrf2 immunohistochemical expression in gastric cancer and its association with clinicopathological features

**Figure 2:**
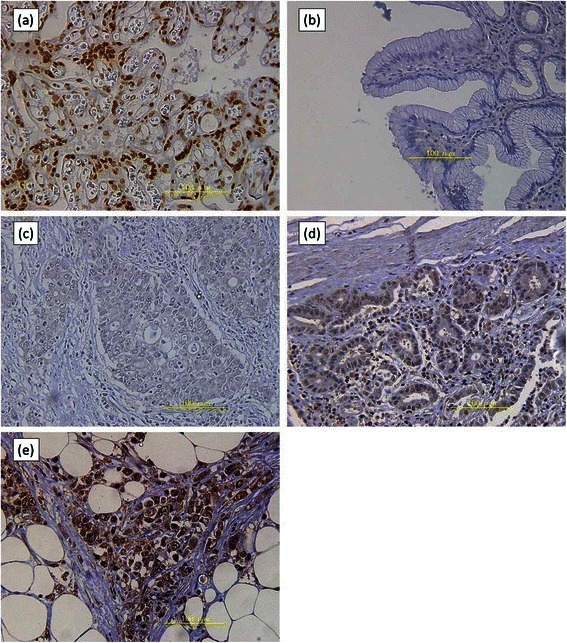
**Immunostaining of nuclear factor (erythroid-2)–related factor 2 (Nrf2) in clinical gastric cancer samples.** Representative immunostaining of Nrf2 in **(a)** Positive control, noncancerous placental tissue; **(b)** normal stomach tissue; **(c – e)** Gastric cancer tissues; **(c)** negative staining of Nrf2, **(d)** weak staining (+1) of Nrf2, **(e)** strong staining (+2) of Nrf2 (original magnification, ×400). Expression of Nrf2 in clinical samples. Immunostaining of Nrf2 (original magnification, ×400)

We next determined the expression and subcellular localization of Nrf2 in 175 paraffin-embedded archival gastric cancer tissues, using immunohistochemistry. The clinicopathological characteristics of the patients are shown in Table 1. Nrf2 immunoreactivity was detected mainly in the nucleus (Figure 2). Further evaluation of nuclear Nrf2 immunoreactivity indicated that the Nrf2 immunoreactive pattern differed between differentiated gastric cancer cells and undifferentiated gastric cancer cells in the same specimen. Thus, undifferentiated gastric cancer cells were more likely to display positive Nrf2 immunoreactivity compared to differentiated gastric cancer cells. Of the 175 patients, 108 (61.7%) displayed an Nrf2 immunohistochemical score of greater than 100 and were classified as Nrf2 positive. The remaining 67 (38.3%) patients were classified as the Nrf2 negative group. Table 2 shows the correlation between Nrf2 expression and clinicopathological features. Nrf2 positive expression was significantly associated with gender, tumor size, tumor depth, lymph node metastases, lymphovascular invasion, histologic classification and clinical stage of gastric cancer.

**Table 1:** **Patient characteristics**

	No.
Gender	
Male	116
Female	59
Age	
Mean	66
Range	31-84
Operation	99
Total gastrectomy	
Distal	56
Proximal	14
Partial	6
Stage	
IA, IB	47
IIA, IIB	33
IIIA, IIIB, IIIC	95
Histology	
Differentiated	71
Undifferentiated	104

Stage was assessed according to 7^th^ edition of tumor node metastasis classification.

**Table 2:** **Correlation between expression of nuclear factor (erythroid-2)–related factor 2 (Nrf2) and clinicopathological factors**

Clinical factors	Expression of Nrf2		P- value
	Negative	Positive	
	n = 67 (38.3%)	n = 108 (61.7%)	
Age			n.s.
< 65	22	47	
≧65	45	61	
Gender			<0.05
male	51	65	
female	16	43	
Tumor size			<0.01
< 5 cm	36	27	
≧5 cm	31	81	
Tumor depth			<0.01
T1a, T1b, T2	31	19	
T3, T4a, T4b	36	89	
Lymph node metastases			<0.05
Yes	35	74	
No	32	34	
Stage			<0.01
IA, IB	28	19	
IIA, IIB	12	21	
IIIA, IIIB, IIIC	27	68	
Lymphovascular invasion			<0.05
Yes	49	90	
No	18	14	
Histology			<0.01
Differentiated	37	34	
Undifferentiated	30	74	

### Nrf2 immunohistochemical expression in gastric cancer and its association with 5FU resistance

Table 3 shows the correlation between Nrf2 expression and 5FU resistance in the 72 patients who were treated with 5FU-based adjuvant chemotherapy. Of these 72 patients, 59 were Nfr2 positive and 13 were Nfr2 negative. Out of the 59 Nfr2 positive patients, 43 (72.9%) patients were 5FU resistant, whereas only 5 (38.5%) of the 13 Nrf2 negative patients were 5FU resistant. The difference between the percentage of Nrf2 positive and Nrf2 negative patients in the 5FU resistance group was statistically significant (p = 0.024).

**Table 3:** **Correlation between expression of nuclear factor (erythroid-2)–related factor 2 (Nrf2) and 5-Fluorouracil (5FU) chemosensitivity**

5FU sensitivity	Expression of Nrf2		P- value
	Negative	Positive	
Resistant	5	43	0.024
Sensitive	8	16	

### Survival analysis

**Figure 3:**
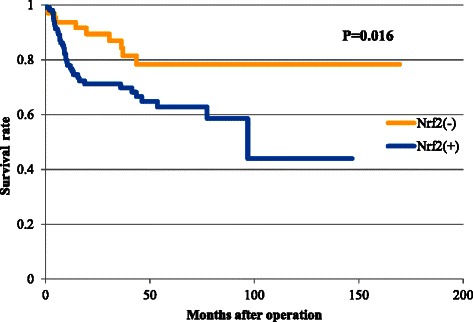
**Postoperative outcomes of 175 gastric cancer patients according to nuclear factor (erythroid-2)–related factor 2 (Nrf2) expression.** Kaplan Meier analysis of the postoperative outcomes of 175 gastric cancer patients according to Nrf2 expression. Survival curves were constructed for the 175 patients that were divided into Nrf2-positive and Nrf2-negative groups. The Nrf2-positive group had a significantly poorer outcome than the Nrf2-negative group (p < 0.01).

Kaplan Meier analysis indicated that the overall survival of the Nrf2 positive group was significantly poorer than that of the Nrf2 negative group (61% vs. 79% respectively) (p < 0.01) (Figure 3).

In addition, tumor depth, tumor size, lymph node metastases, histological classification, lymphovascular invasion were also significant factors for survival based on univariate analysis (Table 4).

**Table 4:** **Univariate and multivariate analyses of prognostic factors in gastric cancer**

Clinical factors	Univariate	Multivariate		
	p	Multivariate P	Hazard ratio	95% confidence interval
Age	0.42	-	-	-
Gender	0.50	-	-	-
Depth	<0.01	0.25	2.44	0.52 - 11.44
Tumor size	<0.01	0.35	1.58	0.61 - 4.09
Nodal involvement	<0.01	0.03	5.02	1.18 - 21.41
Lymphovascular invasion	<0.05	0.27	0.35	0.05-2.26
Histology	<0.05	0.17	1.1.62	0.81 - 3.24
Nrf2	<0.05	0.40	1.37	0.66 - 2.87

Nrf2, nuclear factor (erythroid-2)–related factor 2.

We also performed multivariate analysis of these six factors including Nrf2 as an independent prognostic marker. Lymph node metastases was selected as independent prognostic marker. However, Nrf2 expression was not selected (Table 4).

## Discussion

The antioxidant Nrf2, which functions to protect normal cells from numerous damaging oxidative conditions, has been considered to be a major cellular defense mechanism. However, many papers have indicated the overexpression of Nrf2 and its downstream genes in many types of human cancer [19-25]. These reports showed that Nrf2 not only protects normal cells from transforming into cancer cells, but may also facilitate cancer cell proliferation and prolong survival [18]. The present report is the first report to show an association between the expression level of Nrf2 in the nucleus of gastric cancer cells and clinicopathological findings.

In the current study, Nrf2 immunoreactivity was detected predominantly in the nucleus, not only in vivo but also in vitro in a number of gastric cancer cell lines. It has been speculated that persistent nuclear expression of Nrf2 in gastric cancer cell lines results in the production of antioxidants, which confer on these cancer cell lines a high ability to resist reactive oxygen species (ROS). An similar phenomenon was reported by Ma et al. for cervical cancer tissue They reported that upstaging of cervical cancer leads not only to a higher concentration of Nrf2 in the nucleus of the cancer cells, but also to a higher concentration of downstream antioxidant response proteins [22]. It can therefore be theoretically proposed that the gastric cancer cell lines with nuclear Nrf2 expression would have a higher malignant potential through this mechanism.

We immunohistochemically identified a high rate of Nrf2 expression in gastric cancer clinical specimens (Table 2). Thus, 61.7% of the specimens were Nrf2 positive, which was higher than the percentage of positive specimens reported in non-small cell lung carcinoma (26%) [21] or in gallbladder cancer (23%) [23].

Furthermore, there were significant correlations between Nrf2 expression and several clinicopathological factors such as tumor size, tumor depth, lymphatic invasion, lymph node metastases, and tumor histology. Our findings are in accordance with those of Wang et al. who reported that expression of Nrf2 in gallbladder cancer was significantly associated with differentiation, stage, and lymph node metastases [23].

Recently Xiu-Freg Hu et al. similarly investigated Nrf2 expression in gastric cancer [26]. They also suggested a prognostic significance of Nrf2 expression in accordance with our results. However, they reported that Nrf2 immunoreactivity was detected in the cytoplasm but that it was not detected in the nucleus. This different result may be due to the fact that they used different antibodies from the ones used in the present study. It has been well documented that Nrf2 exerts its antioxidant ability only when it translocates to the nucleus from the cytoplasm [27]. Our immunoblotting analysis showed that Nrf2 was predominantly present in the nucleus of the cells of gastric cancer cell lines. We therefore considered that the persistent overexpression of Nrf2 in the nucleus of gastric cancer cells likely worked as an antioxidant that protected the gastric cancer cells from ROS, and that Nrf2 nuclear expression may reflect aggressive behavior of gastric cancer. Based on this theory, we considered it essential to evaluate nuclear, but not cytoplasmic expression of Nrf2 in clinical specimens of gastric cancer.

In this study, we showed the prognostic value of Nrf2 expression by univariate analysis. Although Nrf2 positivity was not selected as an independent prognostic marker by multivariate analysis, this finding may be explained by the possibility that Nrf2 positivity was significantly affected by nodal involvement which was regarded as poor prognostic factor. Solis et al. and Wang et al. similarly reported that Nrf2 expression was associated with poor overall survival in non-small cell lung carcinoma [21] and in gallbladder cancer [23], respectively. These data suggest that Nrf2 expression might be used as a significant prognostic parameter for prediction of the survival of postoperative gastric cancer patients.

It has recently been reported that high Nrf2 expression may facilitate and prolong cancer cell survival following anticancer chemotherapy and radiation therapy [14,16,19-22,28-33]. Our results also showed that positive Nrf2 expression was significantly associated with resistance to 5FU based adjuvant chemotherapy. These combined results may imply that, by evaluating the expression level of Nrf2 in the nucleus of gastric cancer cells, it might be possible to predict the best candidates who can benefit from receiving not only adjuvant chemotherapy but also neoadjuvant chemotherapy.

Genetic or functional inhibition of Nrf2 has been shown to result in the repression of cellular Nrf2 -regulated antioxidant enzymes, including cellular glutathione, thioredoxin and non-protein thiols. Ultimately, these alterations may restore the sensitivity of cancer cells to anticancer drugs and radiation therapy. Thus, Cho et al. reported that functional inhibition of Nrf2 leads to sensitization of cancer cells to alkylating anticancer agents [31]. Furthermore, Ma et al. reported that the combination of cisplatin and knockdown of Nrf2 dramatically and significantly inhibited tumor growth in vivo [22].

Taking these evidences into account, it is very likely that a new chemotherapeutic protocol that involves Nrf2 regulation will be introduced in the near future. For example, the efficacy of chemotherapy could be first predicted by evaluation of Nrf2 expression and, if any of Nrf2 positive patients are assessed as being resistant to chemotherapy, then it may be possible to achieve sufficient efficacy of chemotherapy by concomitant inhibition of Nrf2. Therefore a new chemotherapeutic protocol that includes antioxidant therapy may be a useful method for solving a clinical problem.

## Conclusions

Nrf2 expression is closely associated with clinicopathological factors and the prognosis of gastric cancer patients. Nrf2 expression in gastric cancer may be useful for evaluation of biological malignant potential, which may be mediated in part by Nrf2 enhancement of the antioxidant ability of gastric cancer cells. Antioxidant therapy might be a promising approach for the treatment of Nrf2 positive gastric cancer patients.

## Abbreviations

Nrf2: NF-E2-related factor 2
OS: Oxidative stress
Keap 1: Kelch-like ECH-associated protein 1
ARE: antioxidant response element
FBS: Fetal bovine serum
PBS: Phosphate buffered saline
TBS: Tris-buffered saline
TBST: TBS-Tween 20
5FU: 5-Fluorouracil
ROS: Reactive oxygen species
